# Enigma of Gastric Teratoma in Infants: A Case Series

**DOI:** 10.1055/s-0044-1800885

**Published:** 2024-12-16

**Authors:** Soumitra Saha, Mayank Tripathi, Kumar Vineet, Ajinkya Kale, Pooja Pande, Zachariah Chowdhury, Raghwesh Ranjan

**Affiliations:** 1 Division of Pediatric Surgical Oncology, Department of Surgical Oncology, Mahamana Pandit Madan Mohan Malaviya Cancer Centre, Varanasi, Uttar Pradesh, India; 2 Tata Memorial Centre, Homi Bhabha National Institute, Mumbai, Maharashtra, India; 3 Department of Surgical Oncology, Mahamana Pandit Madan Mohan Malaviya Cancer Centre, Varanasi, Uttar Pradesh, India; 4 Department of Surgical Oncology, Tata Memorial Hospital, Mumbai, Maharashtra, India; 5 Department of Radiodiagnosis, Mahamana Pandit Madan Mohan Malaviya Cancer Centre, Varanasi, Uttar Pradesh, India; 6 Department of Onco-Pathology, Mahamana Pandit Madan Mohan Malaviya Cancer Centre, Varanasi, Uttar Pradesh, India; 7 Department of Pediatric Oncology, Mahamana Pandit Madan Mohan Malaviya Cancer Centre, Varanasi, Uttar Pradesh, India

**Keywords:** teratoma, stomach, infants, gastric, germ cell tumor

## Abstract

Gastric teratomas are an extremely rare variety of teratomas in children. The aim of our series is to present the natural history and progression of the disease. Retrospective analysis of prospectively maintained data of all the gastric teratoma patients treated at our center was done from their electronic medical records. A total of four cases of gastric teratoma were found to have been treated, all of them being less than 1 year old with three-fourths being male. Typical imaging features of teratoma along with normal germ cell tumor markers helped in making a diagnosis. Surgery is the main form of treatment. In final histopathology, there was equal distribution of mature and immature teratomas. On long-term follow-up, there has been no incidence of recurrence. There needs to be reporting of more cases to verify its natural history.

## Introduction

Teratoma is a type of germ cell tumor that contains all three germ cell layers, that is, ectoderm, mesoderm, and endoderm. It can occur both in children and adults. They are classified as mature and immature teratomas on the basis of the differentiation of their contents. In children, they usually occur at the sacrococcygeal region followed by gonadal, mediastinal, presacral, retroperitoneal, cervical, and intracranial regions in order of decreasing frequency. 1

Gastric teratoma is a rare type of teratoma that accounts for less than 1% of all teratomas occurring in children. It can be benign or malignant. 2 However, they are the most common teratomas of the gastrointestinal tract. 3 In children, they mostly occur in infants. These teratomas have peculiar characteristics like they mostly occur in males and most commonly present as abdominal mass. The syndromic association of gastric teratoma is not well-documented. They are mostly benign in nature. 4 Since gastric teratomas are a rare entity, knowledge about their course of presentation and progression is limited in the available literature. In this report, we are presenting four cases of gastric teratomas trying to bring out their natural history, clinical presentation, management along with prognosis.

## Case Series

A retrospective analysis of prospectively maintained data of all the gastric teratomas patients who were treated at our oncology center between 2020 and 2024 was performed from their electronic medical records. A database related to various characteristics of these patients like their presenting complaint, age of presentation, gender, duration of symptoms, and baseline tumor markers value was developed. Along with these characteristics, their radiological presentation, surgical procedure, the requirement of neoadjuvant or adjuvant chemotherapy, final histopathology report, and follow-up presentations were also included in the database. Radiological images were carefully evaluated by a specialist radiologist to find any distinctive characteristic of gastric teratoma and biopsy was evaluated by an oncopathologist.

In total, four patients were treated at our center. A contrast-enhanced computed tomography (CECT) scan of the thorax, abdomen, and pelvis was performed in all four patients. Preoperative tumor markers (α fetoprotein [AFP], β human chorionic gonadotropin [b hCG], and lactate dehydrogenase [LDH]) were investigated at baseline and were followed postoperatively if they were elevated. A preoperative biopsy was done on two patients. The postoperative follow-up protocol is described in subsequent sections.

Patients' characteristics are presented in Table 1 .

**Table 1:** Patients' characteristics

Case	Sex	Age at presentation	Baseline AFP (ng/ml)	Baseline b hCG (mIU/ml)	Baseline LDH (U/L)	Tumor volume (mL)	Follow-up
Case 1	Male	1 month	357.9	2	272	515	4 years: healthy
Case 2	Male	7 months	22.9	0.3	318	1,643	3 year 6 months: healthy
Case 3	Male	5 months	17,811	2.7	220	1,016	3 years: healthy
Case 4	Female	2 months	2,054.4	2	254	554	1 year: healthy

Abbreviations: AFP, α fetoprotein, b hCG, β human chorionic gonadotropin; LDH, lactate dehydrogenase.

There were four patients with gastric teratoma, out of which one was female and the other three were males. Their age of presentation varied from 1 to 7 months, that is, all of them were infants. In all four patients, their chief presenting complaint was an abdominal lump. In one of the patients, a mass was detected in the antenatal scan. One patient underwent ultrasonography of the abdomen before presenting to our center which showed a multiseptated cystic mass.

**Fig. 1:**
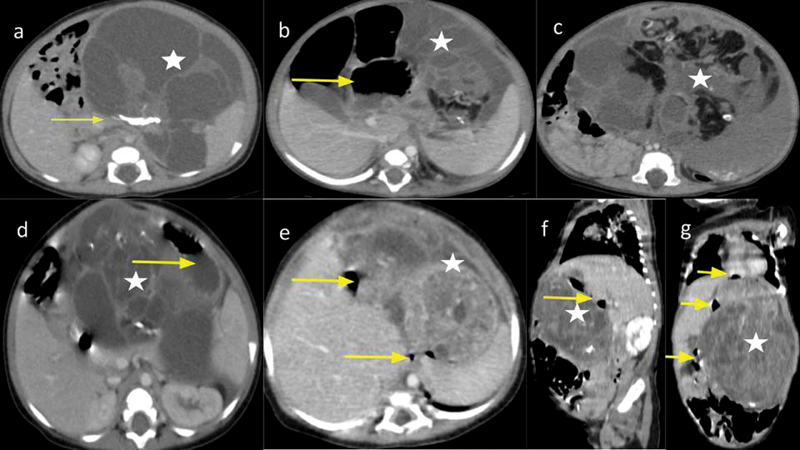
( **a** ) Axial contrast-enhanced CT image showing a predominantly multiseptated cystic mass (star) with heterogeneously enhancing soft tissue arising from the greater curvature of the stomach compressing the oral contrast-containing body of the stomach (yellow arrow). ( **b, c** ) Axial contrast-enhanced CT image showing a soft tissue mass (star) showing areas of fat, fluid, and calcifications within, epicentered in the lesser foramen, and pushing the body of the stomach (yellow arrow) to the right. ( **d** ) Axial contrast-enhanced CT image showing a predominantly septated cystic mass (star) showing areas of soft tissue, fat, and calcifications within, with loss of fat planes with the lesser curvature of the stomach (yellow arrow) causing displacement of the stomach anteriorly and to the left. ( **e** – **g** ) Axial ( **e** ), sagittal ( **f** ), and coronal ( **g** ) sections of contrast-enhanced CT show a heterogeneously enhancing soft tissue mass (star) showing areas within, epicentered in the lesser foramen and compressing the air-filled lumen of the stomach (yellow arrows) with right posterolateral displacement. CT, computed tomography.

As a rule, all four patients had a CECT scan done after presenting to our side. All of them had CECT findings of solid cystic mass with thick septations, and areas of coarse calcifications and fat attenuation ( Fig. 1 ), which is typical of teratomas and clinched diagnosis in its favor. The mean tumor volume of gastric teratomas in this series was 932 mL. Intraoperatively, in three cases, mass was seen arising from the greater curvature of the stomach, and in one case, it was originating from the fundus of the stomach.

One of the patients with gastric teratoma also had a yolk sac component for which he received six cycles of chemotherapy combination of bleomycin, etoposide, and cisplatin, three cycles in the neoadjuvant setting and remaining as adjuvant chemotherapy. The gastric teratoma case with the yolk sac component had a raised baseline AFP value, otherwise baseline values of AFP, b hCG, and LDH were within normal limits according to age in rest of the cases.

**Fig. 2:**
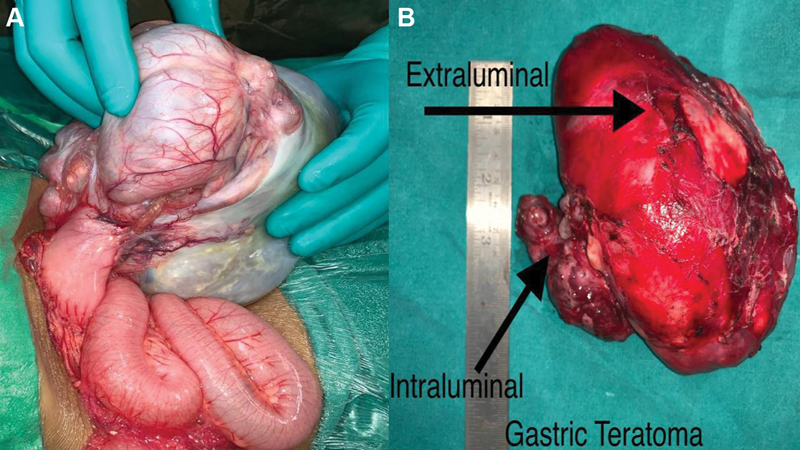
( **a** ) Intraoperative image of teratoma arising from the stomach. ( **b** ) Image of an excised gastric teratoma with both exophytic and endophytic components.

Three patients underwent en bloc excision of the mass with wedge excision of the stomach as the surgical procedure and one of the patients had a partial gastrectomy as the surgical intervention since the mass had both exophytic and endophytic components ( Fig. 2 ). Final histopathology records of two patients were reported as mature cystic teratoma. In one patient, biopsy was reported as grade 1 residual viable immature teratoma. This patient had a yolk sac component in the preoperative biopsy. The remaining patient's biopsy was reported as immature teratoma, grade 3.

## Discussion

Teratomas are a form of germ cell tumors that derive tissues from all three germ cell layers. They are congenital embryonal neoplasms. Morphologically, teratomas are of three types: solid, cystic, and mixed. Teratomas are the most common type of germ cell tumors in children. While they can occur in any part of the body, in adolescents they are most commonly found in gonads. In children, extragonadal teratomas are more common with the sacrococcygeal region being the most common location of origin. The majority of teratomas are seen in females. 1 5

On the basis of differentiation of components, teratomas are divided into mature and immature varieties. Mature teratomas are considered benign tumors. Immature teratomas are histologically characterized by immature cells, mainly neuroepithelial tissues. On the basis of immature neural elements, they are histologically graded by the Norris grading system and grades have an impact on the prognosis. 6 Immature teratomas have malignant potential.

Gastric teratoma is an extremely rare type of teratoma with less than 1% incidence of all infancy and childhood teratomas. Eustermann and Sentry 7 reported the first case of gastric teratoma in 1922. Since the discovery of gastric teratoma, not more than 200 cases of gastric teratomas have been reported in available English medical literature. They are mostly reported as exophytic in nature with a minority of cases being endophytic or mixed exophytic and endophytic type. 7 Although the common site of origin of gastric teratoma is reported on lesser curvature, in our series, 75% of cases have originated from greater curvature. They can be diagnosed on antenatal scans as well. In one of our cases, antenatal scans lead to the diagnosis. As opposed to most teratomas, gastric teratomas are more common in males as can be seen in our series where three (75%) cases were males.

The etiopathogenesis of gastric teratomas is not known and similar to other teratomas they have been proposed to originate from migrated totipotent germ cells. Most gastric teratomas present as abdominal lumps although presentation will also depend on whether they are exophytic or endophytic in nature varying from palpable abdominal lump and gastrointestinal bleeding to gastric outlet obstruction. There have been reports of spontaneous rupture of large gastric teratomas. Large gastric teratomas have also been reported to cause various obstetric problems. 8 Germ cell tumor markers like AFP, b hCG, and LDH value in gastric teratomas are within the normal range as appropriate for that age like other teratomas until and unless they also have other germ cell tumor components. One of our cases also had a yolk sac component and in view of the same, AFP value was raised in that particular patient.

Preoperative diagnosis of gastric teratomas is challenging. There is no gold standard imaging investigation. Most gastric teratomas are predicted on computed tomography (CT) scans of the abdomen and sometimes by ultrasonography of the abdomen, although there are reports of getting X-rays and magnetic resonance imaging of the abdomen done while working up for the diagnosis. 8 Large multiseptated cystic lesions with coarse calcifications and areas of fat attenuation originating from the stomach can work as good predictive radiological markers like other teratomas. Previously mentioned CECT features along with normal tumor marker values can accurately predict gastric teratomas. Upper gastrointestinal endoscopy-guided biopsy or image-guided biopsy can help in establishing a diagnosis but whether preoperative diagnosis will alter the course of management is debatable.

On the basis of clinico-radiological features, there can be many differential diagnoses of gastric teratoma like nephroblastoma, neuroblastoma, gastric gastrointestinal stromal tumor (GIST), rhabdomyosarcoma, liposarcoma, etc. 9

Surgery is the main form of treatment for gastric teratoma with chemotherapy reserved for teratomas with other germ cell components. As most of the teratomas are exophytic in nature, excision of teratoma with a small gastric wedge is required as a surgical procedure in most cases. In the available literature, there have been reports of subtotal gastrectomy to total gastrectomy for the concerned disease. There is no consensus over the minimum margin required during surgery. In our opinion, the presence of negative margins in postoperative biopsy reports is adequate. Other authors also agree with the presence of negative margins. 10

On gross examination, gastric teratomas have both solid and cystic components. In histopathology, teratomas can be of mature variety or immature variety. Mature teratomas have well-differentiated tissue and they are considered benign in nature. Mature teratomas are identified as grade 0. They form the bulk of cases. On the other hand, immature teratomas are graded 1 to 3 depending on neuroglial and neuroepithelial tissues and the degree of mitotic activity. Immature teratomas harbor malignant potential so histopathological examination is cautiously performed to rule out any microfocus of yolk sac components. The presence of other germ cell tumor varieties makes teratomas malignant. 8 In our series, two (50%) cases are of immature type.

Gastric teratoma needs follow-up after surgery. There is one case report of recurrence of gastric teratoma. 11 Currently, there is no follow-up guideline available. At our center, in a multidisciplinary team meeting consensus developed over follow-up protocol. They are followed up with ultrasonography of the whole abdomen and germ cell tumor markers in cases of immature teratoma. In the first year postoperatively, the patients were followed every 3 months. From the second year onwards, a follow-up examination was performed every 6 months till 5 years. Three of our patients have surpassed 3 years of postoperative follow-up and one patient has been followed up for 1 year till now.

According to the available literature on gastric teratomas, the prognosis of this enigma seems to be good and bright. Meta-analysis/Systematic review of available cases is the need of the hour to delve into the depth of gastric teratomas.

All procedures followed were in accordance with the ethical standards of the responsible committee on human experimentation (institutional and national) and with the Helsinki Declaration of 1964 and later versions. Informed consent to be included in the study, or the equivalent, was obtained from all patient's legal guardians.

## Notes

Importance for a Pediatric Surgeon Gastric teratoma is a rare teratoma. It presents mostly as abdominal lump. Surgery with negative margins is the main form of treatment. Malignant gastric teratoma may also require chemotherapy. Postoperative follow ups at regular interval is mandatory.
